# A qualitative study of big data and the opioid epidemic: recommendations for data governance

**DOI:** 10.1186/s12910-020-00544-9

**Published:** 2020-10-21

**Authors:** Elizabeth A. Evans, Elizabeth Delorme, Karl Cyr, Daniel M. Goldstein

**Affiliations:** Department of Health Promotion and Policy, School of Public Health and Health Sciences, University of Massachusetts Amherst, 312 Arnold House, 715 North Pleasant Street, Amherst, MA 01003 USA

**Keywords:** Big data, Opioid epidemic, Public health ethics, Data governance, Qualitative methods

## Abstract

**Background:**

The opioid epidemic has enabled rapid and unsurpassed use of big data on people with opioid use disorder to design initiatives to battle the public health crisis, generally without adequate input from impacted communities. Efforts informed by big data are saving lives, yielding significant benefits. Uses of big data may also undermine public trust in government and cause other unintended harms.

**Objectives:**

We aimed to identify concerns and recommendations regarding how to use big data on opioid use in ethical ways.

**Methods:**

We conducted focus groups and interviews in 2019 with 39 big data stakeholders (gatekeepers, researchers, patient advocates) who had interest in or knowledge of the Public Health Data Warehouse maintained by the Massachusetts Department of Public Health.

**Results:**

Concerns regarding big data on opioid use are rooted in potential privacy infringements due to linkage of previously distinct data systems, increased profiling and surveillance capabilities, limitless lifespan, and lack of explicit informed consent. Also problematic is the inability of affected groups to control how big data are used, the potential of big data to increase stigmatization and discrimination of those affected despite data anonymization, and uses that ignore or perpetuate biases. Participants support big data processes that protect and respect patients and society, ensure justice, and foster patient and public trust in public institutions. Recommendations for ethical big data governance offer ways to narrow the big data divide (e.g., prioritize health equity, set off-limits topics/methods, recognize blind spots), enact shared data governance (e.g., establish community advisory boards), cultivate public trust and earn social license for big data uses (e.g., institute safeguards and other stewardship responsibilities, engage the public, communicate the greater good), and refocus ethical approaches.

**Conclusions:**

Using big data to address the opioid epidemic poses ethical concerns which, if unaddressed, may undermine its benefits. Findings can inform guidelines on how to conduct ethical big data governance and in ways that protect and respect patients and society, ensure justice, and foster patient and public trust in public institutions.

## Background

The opioid epidemic has caused extraordinary numbers of accidental injuries, infectious diseases, and premature deaths, contributing to a historically unprecedented shortening of American life expectancy [1–5]. Massachusetts has been especially impacted [6]. More than 4.4% of the Massachusetts population is estimated to have opioid use disorder (OUD) [7, 8]. In 2017, Massachusetts had an estimated 2,056 opioid overdose deaths [9]. For every fatal overdose, there are about 12 nonfatal overdoses [8, 10]; in Massachusetts, this translates into nearly 65,000 nonfatal overdoses. To address the opioid problem, Massachusetts is using a nationally recognized multi-sectoral public health model [11, 12].

Critical to the Massachusetts’ opioid response is the establishment of the Public Health Data (PHD) Warehouse [8, 13–15]. The PHD Warehouse was created in August 2015 via legislative mandate that empowered the Massachusetts Department of Public Health (MDPH) to monitor opioid-related overdose events [8, 14]. The mandate allows for individual-level linkage of administrative datasets from MDPH and other state agencies. Today, the PHD Warehouse encompasses information from more than twenty sources on all Massachusetts residents aged 11 and older with public or private health insurance, covering > 98% of the state's population [16]. Furthermore, MDPH uses a secure analytic environment to provide access to de-identified data for research purposes [13]. Described in detail elsewhere [13–15], innovative solutions were used to protect data privacy, even beyond the standards set by federal and state law, and to create mechanisms for data sharing. Studies conducted with PHD data over the past five years have been critical to documenting the causes and consequences of the opioid epidemic [7, 17–24].The PHD Warehouse is a ground-breaking essential resource for conducting data-driven public health surveillance, resource allocation, intervention planning, and innovative research [25–27].

From an ethical perspective, the PHD Warehouse raises new, and mostly unconsidered issues. While existing administrative data has been used for more than two decades to study addiction treatment outcomes and costs [28–32], the PHD Warehouse is different in several ways. First, it encompasses most of the Massachusetts adult population, not only individuals with addiction who consented to research. Also, the PHD Warehouse was developed by state mandate, establishing it as a potential public health resource. It was created rapidly as aided by technological innovations and within an emergency response context [13–15]. Finally it was developed without adequate knowledge or input from the general population or people who have been most impacted by the opioid epidemic, i.e., people with OUD and their family or friends. Ethics research on biomedical big data warns that although such data may save lives, when the affected population is excluded from data governance, efforts may also be experienced as harmful, and undermine public trust in government [33–36]. For example, the affected population may perceive big data as infringing on privacy [36–38], perpetuate biases, and be unjust [36, 39, 40].

As Massachusetts works to sustain the PHD Warehouse, and as other states seek to assemble and manage big data on opioid use, guidelines are needed on how to conduct ethical big data governance. We address this gap by exploring stakeholder concerns and perceptions of strategies for uses of big data on opioid use. We conclude by discussing recommendations for future big data governance.

## Methods

### Conceptual framework

We drew on the Kass Public Health Ethics Framework [41] to develop the project. This framework specifies that public health officials should communicate with and involve constituent communities, along with experts, to understand the benefits and risks of strategies to address public health threats. Within this context, we solicited perspectives on the benefits and harms of big data on opioid use as perceived by key stakeholder groups: researchers who conduct analysis of big data on opioid use, gatekeepers of these data, and patient advocates.

### Participants

We interviewed 39 key informants. Researchers were recruited from those who had utilized the PHD Warehouse (i.e., biomedical researchers, clinician-researchers, epidemiologists, data scientists), with priority given to authors of peer-reviewed publications. Big data gatekeepers (i.e., data managers, regulatory specialists, legal counsel, ethicists whose position entails gatekeeper duties) were recruited from MDPH staff who manage the PHD Warehouse and also from local agencies that create county-level big data repositories on opioid use. Patient advocates were recruited from community forums held by peer-led support networks for parents and families coping with opioid overdose. Individuals were invited to participate via flyers distributed at public meetings and direct email outreach.

### Data collection and analysis

A semi-structured 1:1 interview (n = 13) or focus group (n = 4 groups; 2–10 participants per group totaling 26 individuals) was conducted in-person or by teleconference, after which participants completed a socio-demographic questionnaire. Data collection was conducted separately with researchers, gatekeepers, and advocates. The discussion guide included the following topics and prompts. (1) Protect and respect patients and society: What are the concerns regarding using big data on opioid use? To what extent and how should we involve people with OUD and allies in big data governance? (2) Ensure justice: How do we ensure that big data does not further privilege certain groups, or widen existing disparities? Which research topics, questions, or methods should be “off limits”? What phenomena shape opioid use but are not captured in big data? (3) Foster patient and public trust in public institutions: How might big data undermine or strengthen relationships between individuals and public institutions? How do we ensure that the potential harms of big data are outweighed by its benefits? When participants hesitated or expressed uncertainty or disagreement, the facilitator invited participants to share their thoughts, indicating that it was appropriate to disagree with the assumptions of the prompt, and asked probing follow-up questions.

Data were collected in March-December 2019. Each discussion lasted 1.0–2.0 h and was held privately either in-person or by video-conference. Participants were compensated $100. To maintain confidentiality, participants were assured that findings would be anonymized. Interviews were digitally recorded, professionally transcribed, and transcripts were reviewed for accuracy. All procedures were approved by the University of Massachusetts Institutional Review Board.

Using thematic analysis [42, 43], the research team reviewed transcripts and developed codes and their definitions. Two research staff coded each transcript independently in ATLAS.ti (ATLAS.ti 2020, Version 8), and then met with the Principal Investigator to compare and refine their codes, definitions, and themes, and resolve minor discrepancies regarding the relative salience of themes through consensus-building. Each team member identified major themes inductively, identifying analytical categories from the data. The team examined patterns within and across the transcripts and grouped consistent responses along with illustrative quotations. The entire research team reviewed the resulting summary of themes.

### Results

We examine data from a non-random convenience sample (Table 1). More researchers and gatekeepers than advocates had direct experience with big data. However, most participants referenced the broader context of living in an “information era” in which personal data are routinely collected about individuals without explicit knowledge or consent. Participants observed that when institutions inadequately inform individuals about data uses, it engenders feelings of disrespect, inequity, and distrust. In this section, first we summarize participants’ concerns about big data on opioid use and then we present their ideas on the pros and cons of different strategies for big data use.

**Table 1:** Sample characteristics

	Gatekeepers (n = 8)	Researchers (n = 12)	Advocates (n = 19)
Age, mean (sd)	42.9 (9.6)	42.1 (8.8)	51.2 (15.0)
Race/Ethnicity, %			
White	87.5	83.3	73.7
Asian	0.0	16.7	5.3
Latino	12.5	0.0	5.3
Black or African American	0.0	0.0	5.3
Other	0.0	0.0	10.5
Female, %	75.0	33.3	63.2
Education, %			
HS diploma/GED/Trade Vocation	0.0	0.0	26.3
Associate's	0.0	0.0	42.1
College	12.5	0.0	15.8
Masters or professional degree	62.5	0.0	15.8
MD	0.0	58.3	0.0
PhD	12.5	41.7	0.0
Other	12.5	0.0	0.0

### Participants’ concerns

#### Respect

Participants’ concerns about big data on opioid use mostly focused on respect for persons and potential individual-level harms. Big data links together information about individuals as provided by previously distinct data systems. Participants were provided with accurate information about administrative data linkage and the privacy preserving methods that are typically employed, after which participants were asked to share potential concerns. Participants feared that big data could be misused by government or other institutional actors for “bad intentions,” i.e., in ways that infringe on privacy or worsen the health or welfare of individuals with OUD. While most participants understood that big data were anonymized and bound by other safeguards designed to preclude individual-level harms, some nevertheless worried that these data could be used to deny health insurance claims or use of social welfare programs, jeopardize employment, threaten parental rights, or increase criminal justice surveillance, prosecution, and incarceration. Others focused on the potential limitless lifespan of big data which could “permanently mark” individuals as having OUD and thus result in lifelong negative impacts. Furthermore, researchers, many of whom were also clinicians, observed that patient beliefs that these harms could occur, however unlikely, would deter some from seeking healthcare altogether. Participants reported that individuals with OUD generally are not aware of the existence of big data and were concerned that exclusion of patients from big data deliberations further diminishes the connection between public health professionals and the public. Individuals with OUD were perceived to be more vulnerable to data misuse yet among those least likely to do anything about it. Finally, one gatekeeper observed that “…the benefits and harms [of big data] do not accrue to the same person,” drawing attention to the injustices of when those individuals who contribute their information to big data do not themselves directly benefit. For these and other reasons, participants felt it was critical that individuals be informed about potential big data uses, that strict safeguards are observed, and that processes are instituted to ensure that data are used to benefit people with OUD.

#### Equity

Other concerns pertained to the potential for big data to be misused in ways that increase health inequities. Participants noted how some datasets in the big data warehouse come with significant limitations and “baked in bias,” such as ill-defined variables or omitted phenomena, missing data, and an uncertain causal ordering of events. One gatekeeper observed that the opioid epidemic has resulted from systemic inequities yet, given data limitations, we do not examine or address conditions that enable the epidemic, a problem that ultimately contributes to continued health disparities. Participants cautioned that if big data limitations are ignored or mishandled, then results could be incorrect or misinterpreted.

One participant called for better recognition of inherent biases, saying “I think the best approach for ethical data science is the recognition of our biases as human beings…we understand the world through our prejudgment…[we have a] whole set of biases that allow us to work in the world. Now if we say, ‘Well, I'm not biased…data speak for themselves.’ We need to change that idea, right? They don't.”

Participants also highlighted how big data could exacerbate community-level health and social inequities. For example, geographical hotspot maps of opioid overdoses identify certain communities as being especially hard hit by the opioid epidemic. This attention could have negative economic impacts, yet participants felt that with appropriate safeguards the knowledge gained was worth potential community-level harms.

One researcher said about hotspot maps, “…it shines a light on public health needs and perhaps can help to enforce the direction of resources to curtail that problem…if you don't shine a light on it, things will continue the same and…more people will die…so, I'd rather shine a light on the truth of what's happening, but with the hopes that intervention can follow, such that it doesn’t become an ongoing perennial problem….”

Big data’s ability to yield otherwise unavailable insights into place-based OUD prevalence and harms was thought to be critical for avoiding preventable morbidity and premature mortality.

#### Trust

Participants noted that public mistrust of big data is created when it operates outside the awareness of the people being studied.

An advocate said, “I think it's just a matter of trust. Like when you look at the things that happened with Facebook…[people] were doing some unethical things in collecting data and who they were sharing that data with and not telling people that that data was being collected…that's the basis for this mistrust is that it's just this passive thing. You don't even know it's happening…And I share people's concerns…[in] this information era, this isn't just going to magically disappear when we solve this [opioid] problem. This information is still all going to be sitting somewhere. So, how do we make sure…that we don't misuse it moving forward?”.

Others highlighted how public mistrust is worsened when the involved institutions do not adequately interact with individuals to inform and determine how data are collected, managed, and used.

## Summary

Participants’ big data concerns center on potential privacy infringements due to increased profiling and surveillance capabilities, limitless lifespan, and lack of explicit informed consent. Also problematic is the inability of affected groups to influence how big data are used, the potential of big data to increase stigmatization and discrimination of affected communities and groups despite data anonymization, and uses that ignore or perpetuate biases. Next, we present participants’ consideration of data use strategies as mapped to three broad topics, i.e., ideas for using big data in ways that (1) protect and respect patients and society, (2) ensure justice, and (3) foster patient and public trust in public institutions.

### Strategies to protect and respect patients and society

We asked participants for ideas on how to use big data in ways that protect and respect both individuals and also society. Participants discussed perceived pros and cons of better-informed consent processes, but most suggestions emphasized the value of community advisory boards.

#### Informed consent

Participants generally recognized the need for better communication about the purpose and uses of big data. Some focused on adapting consent forms to make it much clearer that if the individual agrees, their data will be added to a big data warehouse. Others, however, felt that while more explicit opt-in procedures might create more informed populations, it might also cause significant selection bias which could, in turn, potentially impede the ability of science to benefit vulnerable populations.

A researcher said, “the drawback is if you somehow had…selection bias about who's deciding to opt-in and then all of a sudden maybe you're leaving out an especially vulnerable group…[that] would have the potential to perpetuate disparities, too…groups who might be…less trustworthy of research or medicine or public health…[would] just say no. And then you miss the most vulnerable groups…And then we all of a sudden have this data that's not representative and then we're making policy decisions that worsen disparities or access or equity…that could be really problematic.”

Others considered whether individuals should be given opportuntities to review their records after being included in big data, or even change or delete data after the fact. Participants felt this option would be impracticable and could lead to unexpected harms. For example, a researcher recalled his clinical experiences to illustrate how doing so could jeopardize the ability of science to determine the truth, saying:

“…when patients ask for…their medical record…it can get really tricky…many patients can get really upset…they can ask for their data to be changed or edited…and sometimes…[in ways] that's actually not consistent with…our professional practice…[for example] I had noted [patient] sexual orientation…and he asked for that to be changed or removed from the record. And that's actually a piece of information that really guides my clinical practice…you could see how people see their data being used for research purposes might take a similar approach and say, ‘Oh, this data says that I have this diagnosis, I don't want that included in there. What if that ever got out?’ And yet, to mask it is to hide the truth. And in research, we really are trying to get as close to the truth as we can. And so, that's the potential tension that I see is that you could move further from the truth if you give people the opportunity to change individual data points, based on their preferences.”

Participants highlighted how the nature and potential risks of big data research are different from clinical trials, explaining that data are gathered whether analyzed for research or not, which contributes to why big data warrants different types of protections and consent processes.

In considering whether individuals should be able to review and alter records, one data researcher said, “…in a randomized trial…if you don't want to be exposed to an intervention or randomized anymore, that is 100% your right. I should be able to say, ‘I'm done right now. I don't want to do this anymore,’ and those are clearly very important protections to have. But when we're looking at observational data, it already exists. However I've decided to label you, as someone with heart disease or someone with opioid use disorder or someone with diabetes, that just exists…it's already been observed. We're not altering anything, and…if you…say, ‘I don't want to be seen as someone with opioid use disorder,’ I understand…but we label things so that we can get answers. So, I think that the biggest con is that we get then even messier data and can't actually answer questions for people.”

Participants equated big data on opioid use with other types of public health records that are used to monitor emerging epidemics, improve healthcare, and protect population health. Participants felt that consent processes should be different for big data that are used for public health rather than for commercial purposes.

An advocate said, “…public health records are really important in maintaining public health…we've maintained…public health data…[to know] where those epidemics are…So, how are we going to know about it if we're not part of the population? …if you want something out of the data, you've got to allow yourself to be part of the data.”

A gatekeeper said, “…having the opt-out [option]…in a commercial setting…is one thing, but essentially this is public health surveillance. And similarly to other things we surveil, you are compelled by government…being honest about that so that people understand and we're transparent, [that] it's not something that you'd be able to opt out of [and this is the case for] legitimate policy reasons, for public health. So, I think that…[whether to offer an opt-out option] may need to be answered differently in different contexts. In the commercial setting, I'm all for it.”

In contrast to the uncertainty of instituting more or different consent processes, participants overwhelmingly endorsed opportunities for “thoughtful conversations.” For example, one researcher said that distrust could be addressed if providers had a “larger conversation [with patients] about how this [big data] gives back to the community, and how it may improve lives.” Participants also proposed that community forums be established to discuss these issues with the public at large and that decision-making incorporate the viewpoints of people with OUD, specifically with community advisory boards.

#### Community advisory boards (CABs)

Participants identified CABs as a potential key component of ethical big data governance. CABs were seen as part of “a new research paradigm” that would entail “more direct involvement of people whose data are being used” and ensure appropriate oversight. Participants felt that individuals with OUD and their families should be involved in “every stage” of research. This included community involvement in developing research questions and hypotheses, specifying inclusion and exclusion criteria, selecting outcomes, interpreting results, and disseminating findings. Comments highlighted how stakeholder involvement should go beyond dissemination such that stakeholder views influence the design and conduct of big data activities. Perceived CAB benefits included the potential to: empower affected populations to identify potential harms and benefits, ensure research priorities are grounded in meeting health and social needs, enable accurate interpretation, and translate findings into salient practices and policies.

At the same time, however, participants shared several expected CAB challenges. Challenges included limited resources needed to form, manage, and sustain CABs and the inability to engage CAB members with sufficient big data expertise. Some participants feared that CABs could influence the conduct or interpretation of big data research in harmful ways. Finally, participants recommended how to optimize CAB utility, which we explore in-depth elsewhere [44].

### Strategies to ensure justice

Participants had several ideas for ensuring that big data on opioid use does not further privilege certain groups or widen existing disparities in health knowledge or practice. Key suggestions were to advance health equity, set off-limit uses, and recognize big data “blind spots.”

#### Health equity

Participants valued health equity and identified processes to ensure that big data are used to achieve it. Suggestions underscored that health equity be a prominent and distinct goal that is integrated into all aspects of big data research. For example, participants called for better training of researchers on how to conduct health equity research and also the use of analytic methods (e.g., sample weights) to enable broader generalization of findings. Many pointed to aspects of data stewardship itself, highlighting how it could be structured to ensure big data are used for health equity. One researcher called for processes to ensure adequate big data access, “…so that people who are interested in disparities can access it and analyze it” and such that a “diversity of perspectives” are represented, including researchers from different institutions who think about opioid use epidemiologically, but also in terms of resource allocation, and in relation to prevention and treatment. Another researcher suggested that big data stewards play “a more proactive role” by being responsible for setting research priorities and reviewing proposals with health equity and disparities in mind, reasoning that “the default of doing nothing has problems…[not] demanding more ‘just’ projects has its own ethical consequences.” Results highlight the prominent and proactive role of big data stewards to safeguard data while also acting to promote its value and utility.

#### Off-limits topics and methods

Participants felt that big data on opioid use should never be used to harm individuals. Using big data for criminal justice purposes was the most commonly identified off-limits use. One participant said, “…if we were using [big data] to identify people who had used illegal drugs and then prosecute them, that would be, to me, a very, very different story…I wouldn’t think is an appropriate use of data sets constructed for research purposes.”

Others felt that certain frames for research results, and related policy implications, should also be off-limits. Speaking about how research has helped to establish understanding of in utero opioid exposure and its relationship to downstream developmental and medical issues, a researcher observed that this is “critical to know” but findings have been:

“framed in the wrong way [by]…lawmakers [who have said], ‘Children who are exposed to opioids in utero do poorly…and therefore, moms who use opioids during pregnancy should be in prison.’ And so, for me it's less about the study questions being off-limits and more about…how the results are framed and the researcher's responsibility to really help policymakers understand the data in the right way.”

Another participant was concerned that big data could be used to perpetuate racial profiling and unjust criminal justice policies, saying.

“I have concerns about what we do with that information…I have concerns about police and racial justice and police going into communities and doing these stop and frisk policies and all kinds of targeting of minorities and killing black people…the use of big health data in the opioid epidemic could exacerbate this type of problem, if used incorrectly.”

In contrast to these perspectives, others felt that given the urgency of the opioid epidemic, and the need for information on how to address it, no research should be off-limits. Participants were careful to endorse a “no off-limits” approach only with appropriate safeguards and ethical review.

One participant explained, “…I don't think that there are…off-limits data [uses], necessarily, as long as we're very thoughtful about maintaining privacy and deidentifying databases and making it so people aren't identifiable…there are some research questions that…shouldn't be approved by IRBs…but I would leave that to individual institutions to determine. I can't think of any specific things that would be…off-limits, in terms of like objectively studying them, particularly when it comes to opioid use disorder.”

Another participant said, “…well, this is the worst epidemic of our times, right? I feel like we need as much information as possible. We need checks and balances, in terms of securities on the data and deidentification of data. But…we need as much information as possible, in order to better understand what's going on and tease apart what factors are most associated with risk and could turn around and start to curb the opioid epidemic.”

Comments generally reflected a desire to balance ethical concerns against the ability to use big data in meaningful ways to resolve a health crisis.

#### Blind spots

Participants identified critical phenomena that shape opioid use but are not captured in big data. Notably, participants felt that the most important “blind spot” is the limited measurement of opioid and other substance use itself. This big data gap was thought to contribute to spurious or confounded results, unjustified conclusions and policy implications, and an inability to concentrate on the upstream causes of OUD.

One researcher said, “The hugest blind spot…is that we have no measure of substance use, right? And that's…fundamental to what we want to do…I know if you got a prescription and I know if you filled the prescription…and I know if you…[had] an adverse event, [but] I have no idea what…substances you're using, what substances you might be combining those with, how you're using them, on what day…And this really presents itself…as an issue…in how we're driving policy related to access to prescription [opioid] medications…and has led to a real pendulum swing that I think is contributing to actually harming folks…people felt like the problem was too many opioids and let's turn off the spigot and things are going to get better. And it's just a tremendous blind spot…it's clear that there are associations between high-risk prescribing and bad outcomes…but there are other features of individuals with high-risk prescribing that have bad outcomes that are not being captured in these data. And lumping [these groups] together [has] led to…sweeping overarching conclusions and policy changes that may not have been justified and have led to harm.”

A gatekeeper said, “…we don't really know when people are using illicit drugs, until they die…we get post-mortem toxicology results, so we can now say, ‘We're seeing this increasing cocaine in deaths,’ but we don't actually know what people are using…we don’t have good sources of information on…what people are actually doing until they die, which is a little bit too late to do anything about it.”

In addition to opioid and other substance use, participants pointed to several other experiences that shape OUD but are simply missing from big data. As we detail elsewhere [45], other blind spots include early life risk factors (e.g., childhood adversity, family factors), socio-economic status and other social status indicators (e.g., homelessness, poverty), social support status, and exposure to contexts that can increase OUD risks (e.g., incarceration, military service). Participants felt that a broader implication of blind spots is an incomplete or biased understanding of the opioid epidemic and limited thinking on how to address it. It was emphasized that these issues, if unaddressed, could maintain an unjust status quo.

One gatekeeper, speaking on the relationship between public trust and govermental transparency, said, “Well, transparency, of course, is the first thing. How are we using it [big data]? Why are we using it? But…[also] recognizing that it's not perfect…there are problems in the data collection as this is a tool that humans use and humans are fallible…we need to be able to think about how to recognize that. I think that's the most important thing…as long as we're ignoring that side of it, we can't ask people to trust us….”

Results point to the need to create institutional processes to reflect on and respond to the complexities, limitations, and uncertainties embedded in big data.

### Strategies to foster patient and public trust in public institutions

Participants considered ways to foster patient and public trust in the institutions that contribute to and manage big data. An overarching aim was to identify ways to ensure that the potential harms of big data are outweighed by its benefits.

#### Citizen science

Participants shared uncertain and divided views about the utility of enabling citizens to directly access and analyze big data on opioid use. A “citizen science” option was considered, i.e., placing data online for public download and analysis. While participants felt that citizen science could increase public buy-in, this potential benefit was weighed against concerns regarding the complicated nature of big data and the lack of needed expertise to understand it and risks of data breaches and inappropriate interpretations. The following comments were made by five different participants.

“…these data sets are really complicated. I started off…doing my own analyses and my own coding…and it quickly became too complex…and it's hard to think about how you–at a real technical level–involve [the public] in the machinery of doing the work.”

“so is it a good idea to have community members [involved in analysis]? Sure. But it's a very complicated science. You just spend all the time trying to explain the use of the data in such a way that respects what's going on and allows people to understand. I don't know.”

“…allowing patients or patient advocacy groups to have unrestricted access to these big data resources, would probably be too far, because if you don’t have the knowledge or experience to even understand what you’re looking at, it’s…not helpful and increases risk associated with data breaches.”

“I go back and forth on this one…there are so many caveats, and people can then misinterpret the information, which could further impact policy…it's fine to have it there [place data online] because it gets buy-in from citizens. But I do really worry about using things in the improper way, and making…lousy interpretations.”

“I'm very wary of people who have no idea what they're talking about and couldn't possibly understand what is involved in analyzing a data set like this, spouting off uninformed opinions and limiting the legitimate progress of science for public health. I mean, despite the apparent democratization of knowledge in the internet age, not everybody has equal expertise and not everybody knows what they're talking about.”

Participants suggested that if big data were made available to citizens, it would be best to first pilot-test mechanisms and also institute safeguards such as releasing only limited datasets along with sufficient documentation and technical support to enable appropriate uses. Although participants were skeptical about the benefits of citizen science, they were clear that opportunities should be available for the public to be involved in other ways.

#### Stewardship responsibilities: safeguards, transparency, and high standards

Participants agreed that the roles and responsibilities of institutions that are charged with creating and managing big data are critical to fostering public trust and ensuring that potential harms are outweighed by benefits. Key data stewardship activities involved safeguards to protect patient privacy and data confidentiality, clear communication on how data will be used for the greater good, and application of high ethical standards.

A gatekeeper said, “…a lot of the so-called ‘ethical concerns’ about harms with data breaches, confidentiality, privacy, they seem to be taken care of with… safeguards….”-

An advocate said, “…it's really critical that whoever is in charge of…managing this data…[consider] what the criteria are…to release this data…[and] realizing what they're putting together here is community-sensitive information…there has to be a return from…the state [Public Health Department], back to us [the public]…to sustain the trust.”

A gatekeeper said, “…transparency…is really important…we say all the time, ‘This isn't ever going to be used for clinical decision-making, because you will never be able to identify that this person who had this trajectory was that person…you can identify groups of people with similar characteristics and change things at…that higher systems level, but…it's really not there to be calling anyone out or trying to find a person.’ And so I think that can be helpful…you have to be really transparent about that….”

Another gatekeeper said, “…people really wanted to know how we were going to use the data…[and] how we were securing the data. Those were the two biggest things. It wasn't really what we were asking for as much as ‘how safe is it?’ and ‘what are you going to use it for?’ So…we were able to say very clearly any data that we would disseminate to the public would be aggregate-level only, and for very thoughtful purposes, and that if it was above and beyond that for any reason, we would have to go back and consult with them.”

A gatekeeper compared public health data use standards to those that are common in social media and other commercial forums, saying “…we [in public health] have a higher standard…with our [big data] warehouse [and]…public health research…I've been presented with examples of [phone] apps…[and] often that data is going somewhere kind of without…the user's knowledge. And…everything that came out with Facebook…really shed a light on the lack of really any policies…It's a conscious decision…to proactively be an ethical steward, in whom the public could have trust. We set the bar high…higher than legally required.”

Comments reflected participants’ recognition of how big data stewardship can minimize harms and ensure that research mitigates, rather than creates or exacerbates, vulnerabilities faced by individuals with OUD. Moreover, transparent processes convey respect for individuals and, by enabling public scrutiny, can help build trust.

#### Public engagement

Other ideas to foster public trust in big data, and maximize its potential benefits, entailed much greater education and engagement with the affected population and their allies, the health and social providers who serve them, and the general public.

For example, a researcher observed that a way to strengthen trust is to “…translate the message from this academic big data study to the community that's affected…making that an effort, and potentially a stated goal, even before you start the study, is…really important. Like ‘how are you going to translate these findings back to the community from which the data's obtained?’” Similarly, a gatekeeper said, “…the more that you can make those goods consumable by different audiences, not only academics or…policymakers, but…[other] audiences that are in the weeds of the programs or…[in] community engagement…that's very important.”

Also important was returning findings to those who contributed data, while also informing them of how their data helps to address the opioid epidemic, and engagement of the affected population in deciding how to communicate findings.

An advocate said, “…you might see some infographic…[for example] ‘there's 120% more overdose deaths in this geographic region than there was this time five years ago’ And…I think people would feel better about it, knowing if they were actually contributing to this data…I'm probably already a part of that data, but no one told me I was…I'd probably feel a heck of a lot better about it knowing that I was.”

A researcher reflected on experiences of sharing research findings with patients, observing that, “…it falls pretty flat…people just sort of really hold their beliefs…they see even my medical advice as a personal belief…there is often this disconnect…[with] science and results that we as a…scientific community hold as valid…it just doesn’t necessarily matter, if the process and the results are not translated in a way that they're palatable to people or in a way that they can digest. And I just don't think we do a very good job of doing that…and unfortunately now we're living in this time where people are…more paranoid than ever. And so…really engaging people in the process more is probably the only way to do it. I just don't think that either researchers or government…can talk their way into convincing people that this is a reasonable thing to do.”

Participants’ comments suggested that public engagement could offer ways to gain public buy-in and ensure that big data uses cohere with public expectations and values.

#### The greater good

A final idea for engendering public trust in big data was to better convey how it is an essential public resource to protect and produce population health. To this end, participants suggested big data media campaigns and public engagement projects. Some suggested that these and other efforts be guided by an ethics board, an entity with a much broader mission than an IRB.

For example, one participant said, “IRBs are responsible for the regulatory aspects…but…there are other elements of ethics…[for example] ‘What kind of people do we become as a result of the information that we're using?’ That takes us away from ‘protection of people’ into ‘who we are as human beings’…a dumbing-down version of it [ethics is]…about just ‘protecting patients, protecting data’ and ‘if we've checked all the boxes, we're done.’ But I like to think about ethics as something a little bit different….”

This focus on communicating broader benefits was expressed by other participants who talked about big data in terms of being used for the “greater good.”

A gatekeeper said that it’s important to make “…a much clearer connection with the greater good…[big data is] fundamental public health…like with vaccine laws and helmet laws…[that] you might not like…but there's a greater good…and more to be learned by having this information than by not.”

## Discussion

### Key findings

Massachusetts is engaged in unsurpassed use of administrative big data on opioid use as routinely provided by health, criminal justice, and social services systems. Informed by the Kass framework, we documented how key stakeholder concerns regarding big data on opioid use are rooted in perceptions of potential privacy infringements due to linkage of previously distinct data systems, increased profiling and surveillance capabilities, limitless lifespan, and lack of explicit informed consent. Also problematic is the inability of affected groups to control how big data are used, the potential of big data to increase stigmatization and discrimination of affected communities and groups despite data anonymization, and big data uses that ignore or perpetuate biases. We also synthesized stakeholder perceptions of different strategies for big data uses.

### Recommendations for big data governance

Implications of our results inform the following recommendations for big data governance (Table 2).

**Table 2:** Recommendations for big data governance

*Narrow the big data divide* Create processes to prioritize health equity Set off-limits topics and methods Recognize blind spots
*Enact shared data governance* Establish community advisory boards
*Cultivate public trust, earn social license* Perform stewardship responsibilities: safeguards, transparency, high standards Educate and involve the public Communicate the greater good
*Refocus ethical approaches*

#### Narrow the big data divide

A key finding is that individuals with OUD may be particularly vulnerable to potential big data misuses (for examples of potential misuses, see section "Off-limits topics and methods"). This population is generally unaware of big data and is excluded from deciding how it is created or used, representing significant asymmetries in big data knowledge and power. Also, this population faces added risks of OUD-related discrimination and stigma, elevated susceptibility to systemic disadvantages, and diminished opportunities to avoid or ameliorate consequent harms. Furthermore, the benefits of big data on opioid use mostly accrue to future generations while any potential harms are borne today. Thus, when considering big data policies and procedures it may be useful to view individuals with OUD as a population whose status warrants added protections to guard against potential harms. It is also important to ensure that big data research mitigates vulnerabilities rather than creates or exacerbates them. Our results indicate that a few places to start are to prioritize health equity, set off-limits topics and methods, and recognize blind spots.

#### Enact shared data governance

Our findings indicate that shared big data governance systems offer ways to protect people with OUD from potential added harms. Other research has suggested that big data co-governance is an ideal rather than a feasible reality [46]. Consistent with this idea, our findings point to Community Advisory Boards as forums for the affected population to have a say in how data about them is gathered, stored, disseminated, and translated. CABs can be used to engage in transparent and collaborative activities, identify and respond to blind spots and other embedded limitations and uncertainties, and appropriately frame findings and policy implications. More broadly, shared data governance enables affected groups to make the most of their own big data resources.

#### Cultivate public trust, earn social license

Results revealed that as big data stewards, governmental public health is responsible for establishing policies and procedures that enable ethical data governance. Essential elements include transparent information on how big data is regulated, protections and potential risks for individuals whose data may be used, governance mechanisms, accountability pathways, and expected public benefits. These activities promote openness to public scrutiny of big data decision-making, processes, and actions. Such transparency demonstrates respect for persons and contributes to the trustworthiness of public institutions, conditions that are necessary for public support of big data [34, 47]. A related next step is to consider how established principles for good data management and stewardship, such as the Findable, Accessible, Interoperable and Reusable (FAIR) Guiding Principles [48], can be adapted to support knowledge discovery and innovation in the uses of big data on opioid use.

Furthermore, our results suggest that it is important to not assume there is social permission for big data activities and this is the case even when individuals have provided consent, a finding reported by other research [49]. Thus, an important role for big data stewards is to elicit public views, concerns, and expectations in relation to big data and make efforts to ensure that uses do indeed align with public expectations and values. Another important role for stewards is to communicate how big data holds the prospect of direct benefits both for individuals with OUD and also the population at large. These activities should be prepared to address how the potential harms and benefits of big data for public health are different from those posed by big data for commercial purposes [50]. Actions such as these can create a sense of commitment among persons with similar interests to share costs and benefits for the greater good. Finally, data stewards can lead efforts to identify key values for guiding how to use big data on opioid use and for making decisions when those values conflict. Ethics frameworks and deliberative balancing approaches [46, 51] provide options for considering salient values and how to minimize potential harms.

#### Refocus ethical approaches

Ethical guidance for big data research has mostly been concerned with protection from presumed harms, consent, and individual control of data uses [52, 53]. While salient, our results indicate that the ideas of respect, equity, and trust are essential for creating guidelines on ethical uses of big data for public health purposes. In addition, it may be more helpful to refocus ethical approaches to give primacy to community engagement, which extends concerns beyond the individual [52–54].

### Limitations and strengths

Findings are based on a non-random convenience sample of 39 individuals in Massachusetts who are knowledgeable about or interested in big data. Small sample sizes are typical in qualitative research and are not meant to support generalizations, but rather provide depth of information [55, 56]. Our list of concerns and data governance recommendations is not exhaustive, but rather highlights selected points as identified by participants. Researchers and gatekeepers had more direct experience than advocates with big data collection, management, and analysis which likely contributed to variation by group in responses to certain prompts. For example, gatekeepers and researchers shared more than advocates in relation to data stewardship and blind spots whereas advocates shared more in relation to off limits topics and the value of educating and informing the public. We highlight findings that represent overall views. We did not analyze variation in perspectives by group, pointing to an area for future research. Findings pertain to static cross-sectoral administrative big data that is created by and for public health. We do not consider issues that may be unique to big data that is assisted by artificial intelligence or the “internet of things” (e.g., mobile phones, environmental sensors, wearable devices), mined in real-time, or created for commercial, criminal justice, or other uses. A strength is that we solicited perspectives from diverse stakeholder groups, including advocates most impacted by OUD, and regarding big data on opioid use, thereby examining topics that previously have been little studied [57]. Also, the study is set in Massachusetts, which is on the forefront of using big data for public health. We employed qualitative methods to explore the experiences of advocates, researchers, and gatekeepers, thereby gaining insight into the complex set of factors that shape views. Finally, the Massachusetts PHD warehouse originated in the opioid epidemic. However, the PHD warehouse was intentionally designed to be used to study other emergent public health issues and, as such, it is now being used by MDPH to understand maternal and child health inequities and assess the impact of COVID-19 [58]. We expect that similar issues as we have identified in this paper in relation to the opioid epidemic are likely to arise in these other fields of investigation. In this sense, our recommendations for big data governance may generalize to these and other areas where public health big data are used for research purposes.

## Conclusion

Using big data to address the opioid epidemic poses significant ethical concerns that, if unaddressed, may undermine its benefits. Findings can inform guidelines on how to conduct ethical big data governance and in ways that protect and respect patients and society, ensure justice, and foster patient and public trust in public institutions.

## Data Availability

Not applicable.

## Abbreviations

OUD: Opioid use disorder
PHD: Public health data warehouse
MDPH: Massachusetts Department of Public Health
CAB: Community advisory board

## References

[1] Case A, Deaton A (2015). Rising morbidity and mortality in midlife among white non-Hispanic Americans in the 21st century. Proc Natl Acad Sci USA..

[2] Hedegaard H, Warner M, Miniño AM. Drug overdose deaths in the United States, 1999–2016. Hyattsville, MD. National Center for Health Statistics; 2017. 8 p. Report No.: 294.

[3] Kochanek KD, Murphy SL, Xu JQ, Arias E. Mortality in the United States, 2016. Hyattsville, MD. National Center for Health Statistics; 2017. 8 p. Report No.: 293.

[4] Murphy SL, Xu JQ, Kochanek KD, Arias E. Mortality in the United States, 2017. Hyattsville, MD. National Center for Health Statistics; 2018. 8 p. Report No.: 328.

[5] Scholl L, Seth P, Kariisa M, Wilson N, Baldwin G. Drug and opioid-involved overdose deaths—United States, 2013–2017. MMWR Morb Mortal Wkly Rep. 2019;67:1419–1427. doi:10.15585/mmwr.mm675152e1 10.15585/mmwr.mm675152e1 PMC6334822 30605448

[6] Kiang MV, Basu S, Chen J, Alexander MJ (2019). Assessment of changes in the geographical distribution of opioid-related mortality across the United States by opioid Type, 1999–2016. JAMA Netw Open.

[7] Barocas JA, White LF, Wang J, Walley AY, LaRochelle MR, Bernson D (2018). Estimated prevalence of opioid use disorder in Massachusetts, 2011–2015: a capture-recapture analysis. Am J Public Health.

[8] MDPH. An assessment of fatal and non-fatal opioid overdoses in Massachusetts (2011–2015). Massachusetts: MDPH; 2017 Aug. 105 p. Available from: https://www.mass.gov/doc/legislative-report-chapter-55-opioid-overdose-study-august-2017/download. Accessed 28 May 2020.

[9] MDPH. Data Brief: Opioid-Related Overdose Deaths among Massachusetts Residents. Massachusetts: MDPH; 2019 Feb. 4 p. Available from: https://www.mass.gov/lists/current-opioid-statistics#updated-data-q4-2018-as-of-february-2019. Accessed 28 May 2020

[10] Darke S, Mattick RP, Degenhardt L (2003). The ratio of non-fatal to fatal heroin overdose. Addiction.

[11] Formica SW, Apsler R, Wilkins L, Ruiz S, Reilly B, Walley AY (2018). Post opioid overdose outreach by public health and public safety agencies: exploration of emerging programs in Massachusetts. Int J Drug Policy.

[12] Rudder M, Tsao L, Jack HE (2016). Shared responsibility: Massachusetts legislators, physicians, and an act relative to substance use treatment, education, and prevention. AMA J Ethics.

[13] Land T, Bernson D, Hood M, Scurria Morgan E, Andrews BK, Cocchi M, et al. Building a prototype of a statewide public health data warehouse: Data privacy and security issues addressed by the Massachusetts Chapter 55 Opioid Initiative. 2018. In Review.

[14] MDPH. An assessment of opioid-related deaths in Massachusetts (2013–2014). Massachusetts: MDPH; 2016. 96 p. Available from: https://www.mass.gov/doc/legislative-report-chapter-55-opioid-overdose-study-september-2016/download

[15] Land , Scurria Morgan E, Bernson D, Hood M, Andrews BK, St. Clair H, et al. Developing a collaborative approach to addressing critical public health issues: a case study of the Massachusetts Chapter 55 Opioid Initiative. 2018. In Review.

[16] Evans E, Delorme E, Harrington C. The Massachusetts Public Health Data (PHD) Warehouse: History, Current Operation, and Impacts. Report submitted to the Massachusetts Department of Public Health (MDPH). (2019).

[17] Chatterjee A, Larochelle MR, Xuan Z, Wang N, Bernson D, Silverstein M (2019). Non-fatal opioid-related overdoses among adolescents in Massachusetts 2012–2014. Drug Alcohol Depend.

[18] Jasuja GK, Ameli O, Miller DR, Land T, Bernson D, Rose AJ, Berlowitz DR, Smelson DA (2018). Overdose risk for veterans receiving opioids from multiple sources. Am J Manag Care.

[19] Larochelle MR, Bernson D, Land T, Stopka TJ, Wang N, Xuan Z (2018). Medication for opioid use disorder after nonfatal opioid overdose and association with mortality: a cohort study. Ann Intern Med.

[20] Larochelle MR, Stopka TJ, Xuan Z, Liebschutz JM, Walley AY (2019). Medication for opioid use disorder after nonfatal opioid overdose and mortality. Ann Intern Med.

[21] Rose AJ, Bernson D, Ho Chui KK, Land T, Walley AY, LaRochelle MR (2018). Potentially inappropriate opioid prescribing, overdose, and mortality in Massachusetts, 2011–2015. J Gen Intern Med.

[22] Rose AJ, McBain R, Schuler MS, LaRochelle MR, Ganz DA, Kilambi V (2019). Effect of age on opioid prescribing, overdose, and mortality in Massachusetts, 2011 to 2015. J Am Geriatr Soc.

[23] Schiff DM, Nielsen T, Terplan M, Hood M, Bernson D, Diop H (2018). Fatal and nonfatal overdose among pregnant and postpartum women in Massachusetts. Obstet Gynecol.

[24] Stopka TJ, Amaravadi H, Kaplan AR, Hoh R, Bernson D, Ho Chui KK (2019). Opioid overdose deaths and potentially inappropriate opioid prescribing practices (PIP): a spatial epidemiological study. Int J Drug Policy.

[25] KPMG Government Institute. The opioid epidemic: data driven case studies [Internet]. 2017. Available from: https://institutes.kpmg.us/content/dam/institutes/en/government/pdfs/2017/opioid-data-case-studies.pdf. Accessed 28 May 2020

[26] Saloner B, Bachhuber M, Barry CL, Krawczyk N, Pasha O, Sen AP, et al. A blueprint for transforming opioid use disorder treatment in Delaware. Delaware Department of Health and Social Services: John Hopkins Bloomberg School of Public Health; 2018. 33 p. Available from: https://dhss.delaware.gov/dhss/files/johnshopkinsrep.pdf. Accessed 28 May 2020

[27] Smart R, Kase CA, Meyer A, Stein BD. Data sources and data-linking strategies to support research to address the opioid crisis: final report. US Department of Health and Human Services: RAND Corp; 2018 Sep. 102 p. Report no.: EP-67716. Available from: https://www.rand.org/pubs/external_publications/EP67716.html. Accessed 28 May 2020

[28] Anglin MD, Jaffe A, Nosyk B, Urada D, Evans E (2013). Offender diversion into substance use disorder treatment: the economic impact of California's Proposition 36. Am J Public Health.

[29] Ettner SL, Huang D, Evans E, Ash DR, Hardy M, Jourabchi M (2006). Benefit-cost in the California treatment outcome project: does substance abuse treatment "pay for itself?”. Health Serv Res.

[30] Evans E, Grella C, Murphy D, Hser YI (2010). Using administrative data for longitudinal substance abuse research. J Behav Health Serv Res.

[31] Krebs E, Enns B, Evans E, Urada D, Anglin MD, Rawson RA, et al. (2018). Cost-effectiveness of publicly funded treatment of opioid use disorder in California. Ann Intern Med. 2018;168(1):10–19. doi: 10.7326/M17-0611. 10.7326/M17-0611 29159398

[32] Weissman MM (2020). Big data begin in psychiatry. JAMA Psychiatry.

[33] Adjekum A, Ienca M, Vayena E (2017). What is trust? Ethics and risk governance in precision medicine and predictive analytics. OMICS.

[34] Aitken M, de St JJ, Pagliari C, Jepson R, Cunningham-Burley S (2016). Public responses to the sharing and linkage of health data for research purposes: a systematic review and thematic synthesis of qualitative studies. BMC Med Ethics.

[35] Evans BJ. Power to the people: data citizens in the age of precision medicine. Vanderbilt J Entertain Technol Law. 2017 Winter;19(2):243–265. PMC5673282 29118898

[36] Mittelsadt BD, Floridi L (2016). The ethics of big data: current and foreseeable issues in biomedical contexts. Sci Eng Ethics.

[37] De Lusignan S, Liyanage H, Di Iorio CT, Chan T, Liaw ST (2016). Using routinely collected health data for surveillance, quality improvement and research: Framework and key questions to assess ethics, privacy and data access. J Innov Health Inform.

[38] Xafis V (2015). The acceptability of conducting data linkage research without obtaining consent: lay people's views and justifications. BMC Med Ethics.

[39] O’Neil C (2016). Weapons of math destruction: How big data increases inequality and threatens democracy.

[40] Zhang X, Pérez-Stable EJ, Bourne PE, Peprah E, Duru OK, Breen N (2017). Big data science: opportunities and challenges to address minority health and health disparities in the 21st century. Ethn Dis.

[41] Kass NE (2001). An ethics framework for public health. Am J Public Health.

[42] Braun V, Clarke V (2006). Using thematic analysis in psychology. Qual Res Psychol.

[43] Braun V, Clarke V (2014). What can "thematic analysis" offer health and wellbeing researchers?. Int J Qual Stud Health Well-being.

[44] Delorme E, Cyr K, Evans E. Community advisory boards and big data governance: Lessons learned from the opioid epidemic in Massachusetts. Submitted for presentation at the 2020 American Public Health Association Annual Meeting.

[45] Shaffer PM, Delorme E, Achuck E, Cyr K, Evans E. Utility of big data for research on the current opioid epidemic. Submitted for presentation at the 2020 American Public Health Association Annual Meeting.

[46] Xafis V, Schaefer GO, Labude MK, Brassington I, Ballantyne A, Lim HY (2019). An ethics framework for big data in health and research. Asian Bioethics Rev.

[47] Richards NM, King J. Big data ethics. Wake Forest Law Review. 2014 May 19. Available from: https://papers.ssrn.com/sol3/papers.cfm?abstract_id=2384174.

[48] Wilkinson MD (2016). The FAIR guiding principles for scientific data management and stewardship. Sci Data.

[49] Kalkman S, van Delden J, Banerjee A, Tyl B, Mostert M, van Thiel G (2019). Patients' and public views and attitudes towards the sharing of health data for research: a narrative review of the empirical evidence. J Med Ethics.

[50] Zuboff S (2019). The Age of Surveillance Capitalism: The Fight for a Human Future at the New Frontier of Power.

[51] Laurie GT (2019). Cross-sectoral big data: the application of an ethics framework for big data in health and research. Asian Bioethics Rev.

[52] Ballantyne A (2019). Adjusting the focus: a public health ethics approach to data research. Bioethics.

[53] Salerno J, Knoppers BM, Lee LM, Hlaing WM, Goodman KW (2017). Ethics, big data and computing in epidemiology and public health. Ann Epidemiol.

[54] Kahn JP, Mastroianni AC, Sugarman J (2018). Beyond consent: seeking justice in research.

[55] Curtis S, Gesler W, Smith G, Washburn S (2000). Approaches to sampling and case selection in qualitative research: examples in the geography of health. Soc Sci Med.

[56] Creswell JW, Creswell JD (2018). Research design: qualitative, quantitative, and mixed methods approaches.

[57] Rosen DL, Buchbinder M, Juengst E, Rennie S (2020). Public health research, practice, and ethics for justice-involved person in the big data era. Am J Public Health.

[58] Bharel M, Bernson D, Averbach A. Using data to guide action in response to the public health crisis of opioid overdoses. NEJM Catalyst. 2020. 1(5). doi:10.1056/CAT.19.1118

